# Nutritional Interventions for Perimenopausal Anxiety and Depression Targeting Tryptophan and GABA Pathways: A Narrative Review

**DOI:** 10.3390/nu18132185

**Published:** 2026-07-05

**Authors:** Huiying Zhao, Wei Wu

**Affiliations:** 1 School of Exercise and Health, Shanghai University of Sports, Shanghai 200438, China; zhaohy1237@163.com; 2 School of Athletic Performance, Shanghai University of Sports, Shanghai 200438, China

**Keywords:** perimenopause, gut-brain axis, nutrition, anxiety, depression

## Abstract

This narrative review examines perimenopause as a critical transitional phase in women’s lives, often accompanied by elevated vulnerability to anxiety and depression. Dysfunction of the gut–brain axis is one of the key factors contributing to perimenopausal mood disorders and is currently receiving extensive attention. GBA dysfunction can trigger neurotransmitter metabolic imbalance, intestinal barrier impairment, and neuroinflammatory responses. Tryptophan (Trp) and γ-aminobutyric acid (GABA) serve as essential precursors and direct modulators of key neurotransmitters, and the dysregulation of their metabolic pathways has been implicated in perimenopausal anxiety and depression in animal models and limited clinical observations. Trp influences 5-hydroxytryptamine (5-HT) by affecting emotional states. GABA is the primary inhibitory neurotransmitter in the central nervous system and is closely associated with anxiety and depression. Fluctuations in estrogen levels during perimenopause significantly alter the composition and metabolic activity of the gut microbiota, which in turn affects Trp metabolism and GABA synthesis through increased intestinal permeability, activation of immune-inflammatory responses, and disruption of hypothalamic–pituitary–adrenal (HPA) axis function. Although traditional hormone replacement therapy and pharmacological treatments are effective, they are associated with some side effects. Preliminary evidence from in vitro and animal studies suggests that nutritional interventions targeting Trp and GABA metabolism within the gut–brain axis may offer a novel research direction, though their efficacy in perimenopausal women remains to be established. Potential nutritional strategies, including supplementation with Trp and its precursors, inhibition of the kynurenine pathway (KP), and supplementation with probiotics and prebiotics, can modulate Trp and GABA metabolism. This review focuses on Trp and GABA metabolic regulation via the gut–brain axis to explore pathogenesis of perimenopausal anxiety and depression and summarize potential nutritional intervention targets, thereby providing a scientific basis for emotional management in perimenopausal women.

## 1. Introduction

Perimenopause is the transitional stage from the decline of ovarian function to one year after menopause, typically occurring between the ages of 45 and 55. The perimenopausal period is usually categorized as the early menopausal transition period, the late menopausal transition period, and the 12 months after menopause [1]. During the early menopausal transition, menstrual cycles become irregular and frequent. Ovarian feedback to the pituitary gonadotropins, follicle-stimulating hormone (FSH), and luteinizing hormone (LH), is reduced, leading to shorter follicular phases, fewer ovulations, and decreased progesterone production [2]. Estrogen levels show a fluctuating, gradual decline. Changes in FSH levels were used as a reference to determine when a woman in late menopausal transition officially entered menopause [3]. This was indicated when FSH levels reached or exceeded 25 U/L after the last menstrual period, and the length of the menstrual cycle was 60 days or more [4]. This profound endocrine shift not only triggers classic somatic symptoms such as hot flashes and insomnia, but also significantly increases the risk of anxiety and depression [5]. The current thinking is that anxiety and depression are not discrete categories but rather have common biological underpinnings and may form at least part of a continuum or affective disorder spectrum [6]. In the present review, the terms ‘depressive’ and ‘anxiety’ refer to subclinical emotional fluctuations or psychological distress commonly reported during perimenopause, rather than formal categorical diagnoses of psychiatric conditions (such as Major Depressive Disorder or Generalized Anxiety Disorder). Epidemiological data indicate that perimenopause is a high-incidence period for mood disorders, with the prevalence of depression reaching 15–50% and the prevalence of anxiety significantly higher than that in women of reproductive age [7]. Anxiety and depression not only severely impair women’s quality of life, but are also closely associated with long-term health risks [8]. The management of perimenopausal symptoms requires distinction of the target. Hormone replacement therapy (HRT) is a clinical treatment for vasomotor symptoms, but it is not the preferred option for depression or anxiety [9]. The NICE guidelines recommend menopause-specific cognitive behavioral therapy as an independent option, applicable when HRT is contraindicated or when patients prefer non-hormonal intervention; diagnosed depression or anxiety disorders should follow the standard psychiatric treatment pathway, including antidepressants and psychotherapy [10]. It is worth noting that NICE does not recommend SSRIs/SNRIs or clonidine as the first-line treatment for purely vasomotor symptoms. Medical professionals should tailor treatment based on clinical factors and patient preferences. They should conduct cardiovascular and breast cancer risk screenings for women before initiating menopausal hormone therapy and recommend the most appropriate treatment plan based on the risks and benefits. Nutritional intervention has been regarded as a potential supplementary or complementary strategy in recent years. Therefore, exploring safe and effective non-pharmacological intervention strategies to improve the emotional health of perimenopausal women has become a major focus of current research.

In recent years, the gut–brain axis in emotional regulation has garnered extensive attention. The gut–brain axis is a complex bidirectional communication system involving intricate interactions among the gut microbiota, the immune system, and the nervous system [11]. Tryptophan (Trp) and γ-aminobutyric acid (GABA) serve as essential precursors and direct modulators of key neurotransmitters, representing critical signaling molecules within the gut–brain axis. The dysregulation of their metabolic pathways is considered a pivotal mechanism underlying perimenopausal anxiety and depression. 5-hydroxytryptamine (5-HT) is a core neurotransmitter regulating emotion, sleep, and appetite, with Trp serving as its sole precursor; the metabolic balance of Trp directly influences emotional states [12]. GABA is the primary inhibitory neurotransmitter in the central nervous system, and its reduced levels are closely associated with anxiety and depression [13]. GABA, as a crucial signaling molecule, connects intestinal barrier function, immune homeostasis, and central emotional processing. A review by Lambiase C et al. indicates that GABA improves irritable bowel syndrome by covering interactions within the central nervous system circuitry, at the intestinal level, and the microbiota–intestinal–brain axis [14]. A study conducted by Lambiase C et al. revealed that GABA treatment was more effective than placebo in improving irritable bowel syndrome and was also more beneficial in enhancing emotional health [15]. Notably, gut microbiota can directly synthesize GABA and modulate receptor function. Fluctuations in estrogen levels during perimenopause significantly alter the composition and metabolic activity of gut microbiota, leading to reduced microbial diversity, depletion of specific bacterial taxa that have been associated with SCFA production, neurotransmitter metabolism, or anti-inflammatory functions, including certain Lactobacillus and Bifidobacterium strains, and exhaustion of short-chain fatty acid (SCFA)-producing taxa. However, it is worth noting that due to differences in geographical location, dietary patterns, body mass index, drug exposure, sequencing methods, and specific menopausal stages, the microbial composition characteristics in each research cohort will vary. This gut dysbiosis affects Trp metabolism and GABA synthesis through increased intestinal permeability, activation of immune-inflammatory responses, and hypothalamic–pituitary–adrenal (HPA) axis disruption, ultimately impacting brain emotional states. Therefore, nutritional interventions targeting Trp and GABA metabolism within the gut–brain axis offer a novel perspective for the treatment of perimenopausal anxiety and depression. This review systematically examines this topic from the perspectives of epidemiology, pathological mechanisms, potential nutritional intervention strategies, and future research directions.

To maintain methodological rigor and transparency, a comprehensive literature search was executed across electronic databases including PubMed, Web of Science, and Embase from database inception up to early 2026. Search terms were structured around combinations of Boolean operators and keywords: (‘perimenopause’ OR ‘menopausal transition’ OR ‘menopause’) AND (‘anxiety’ OR ‘depression’ OR ‘mood disorders’) AND (‘gut-brain axis’ OR ‘microbiota’ OR ‘microbiome’) AND (‘tryptophan’ OR ‘kynurenine’ OR ‘GABA’ OR ‘gamma-aminobutyric acid’). This paper is framed as a comprehensive narrative review. Peer-reviewed clinical trials, observational studies, animal models, and in vitro mechanistic papers were evaluated. Where perimenopause-specific evidence was unavailable, evidence from broader adult populations or experimental models was discussed separately.

## 2. Perimenopausal Anxiety and Depression

### 2.1. Epidemiology and Disease Burden

Perimenopause represents a vulnerable window of profound physiological and psychological fluctuation in women’s lives, during which the prevalence of mood disorders such as depression and anxiety increases significantly. A cross-sectional study of 1146 perimenopausal women aged 45–55 years in Turkey reported a prevalence of depression of 27.1%, with severe, moderate, and mild depression accounting for 3.8%, 12.7%, and 10.6%, respectively [16]. A systematic review and meta-analysis encompassing 102 studies and over 1.14 million women demonstrated that the prevalence of depression in perimenopausal women was 32%, anxiety was 29%, and insomnia symptoms were as high as 42% in postmenopausal women [7]. A survey of 396 menopausal women in Saudi Arabia revealed that 65.2% reported depression and 52.02% reported anxiety [17]. Clinical presentation of perimenopausal anxiety and depression often differs from typical depression, exhibiting atypical characteristics. A clinical study of 90 hospitalized patients showed that perimenopausal patients predominantly presented with atypical depression (63.4%) and anxious depression (87.8%), whereas early postmenopausal patients more commonly exhibited melancholic depression (59.2%) [18]. A network analysis identified irritability as a core symptom of perimenopausal syndrome [19]. Additionally, perimenopausal women are more prone to nonspecific symptoms such as fatigue and somatic pain, which are frequently attributed to menopause itself, resulting in high rates of misdiagnosis and missed diagnosis of anxiety and depression [20]. Collectively, these data indicate that perimenopausal anxiety and depression have become a public health concern that cannot be ignored.

Perimenopausal anxiety and depression not only severely impair women’s emotional states, but also exert profound and multidimensional negative impacts on their social functioning, work capacity, and life satisfaction. A cross-sectional study demonstrated a significant direct negative correlation between psychological distress and health-related quality of life, with menopausal symptoms serving as a partial mediator; notably, emotional problems further deteriorated quality of life by exacerbating somatic symptoms [21]. Furthermore, the synergistic effect of psychological resilience and family support is crucial for protecting perimenopausal women from psychological distress, with mismatches between these factors associated with more severe distress [22]. Long-standing anxiety and depression are closely linked to multiple chronic diseases, significantly increasing long-term health risks for perimenopausal women. Research has confirmed that perimenopausal depression and anxiety are significantly associated with cardiovascular disease, metabolic syndrome, osteoporosis, and cognitive decline. A narrative review of women with type 1 diabetes highlighted that perimenopausal women face elevated risks of osteoporosis, cardiovascular disease, psychological distress, metabolic deterioration, and sexual dysfunction, with estrogen deficiency exacerbating insulin resistance, dyslipidemia, and vascular dysfunction [23].

### 2.2. Hormones with Anxiety and Depression

The decline in ovarian function during perimenopause leads to fluctuating decreases in estrogen levels, representing one of the core driving factors in anxiety and depression. Estrogen exerts significant neuroprotective effects and can regulate 5-HT, norepinephrine, and dopamine, thereby directly influencing emotional states [24]. During perimenopause, the dramatic fluctuations in estrogen levels—rather than a simple decline—are considered the critical mechanism underlying anxiety and depression. Research has proposed that women exhibit distinct sensitivity phenotypes to estradiol (E2) changes, including sensitivity to E2 elevation, E2 withdrawal, bidirectional changes, and insensitivity; these phenotypes determine the timing and severity of depression [25]. A randomized controlled trial further confirmed that perimenopausal women with greater baseline sensitivity to E2 fluctuations showed more significant improvement in anxiety following transdermal estradiol treatment, suggesting that individualized hormone sensitivity serves as an important predictor of therapeutic efficacy [26]. Additionally, estrogen modulates HPA axis function through estrogen receptor β, thereby reducing the organism’s stress response. The decline in estrogen levels during perimenopause results in HPA axis hyperactivation and elevated cortisol levels, consequently triggering anxiety and depression [27].

Beyond estrogen, the decline in progesterone levels and the reduction in its neuroactive metabolites also play a pivotal role in the pathogenesis of anxiety and depression. Allopregnanolone, a metabolite of progesterone, is a potent positive allosteric modulator of GABA-A receptors capable of enhancing the inhibitory function of the GABAergic system and producing sedative and anxiolytic effects. The decrease in progesterone levels during perimenopause directly leads to reduced allopregnanolone, thereby weakening the inhibitory function of the GABAergic system, increasing neuronal excitability, and promoting the occurrence of anxiety [28]. This imbalance in neurotransmitter systems constitutes an important neurobiological basis for perimenopausal anxiety and depression. Clinical studies have also confirmed that the proportion of anxious depression among perimenopausal women is as high as 87.8%, which is closely related to the increased neuronal excitability resulting from diminished GABAergic system function [18].

Furthermore, the synergistic interaction between estrogen and progesterone warrants attention. One study found that elevated ratios of testosterone to estradiol were associated with more severe depression, suggesting that the balance among sex hormones may be more predictive of mood disorder risk than individual hormone levels alone [29]. Therefore, the fluctuating decline of estrogen combined with the reduction in progesterone and its metabolites during perimenopause jointly affects neurotransmitter systems, HPA axis function, and the balance of neuronal excitability, thereby exacerbating anxiety and depression.

## 3. Gut–Brain Axis and Emotional Regulation

### 3.1. Structure and Function of the Gut–Brain Axis

The gut–brain axis is a bidirectional communication network, and its core function is the maintenance of organismal homeostasis and integration of peripheral and central signals [30]. Within this complex network, gut microbiota serve as regulators, profoundly influencing brain function through neural, endocrine, immune, and metabolic pathways [31]. Specifically, gut microbiota can produce various neuroactive metabolites, such as SCFAs, Trp metabolites, and GABA, which can directly or indirectly act on the central nervous system to modulate emotion and behavior [32]. Additionally, gut microbiota can indirectly regulate neuroinflammatory states and participate in the pathogenesis of anxiety and depression by modulating intestinal barrier function and the immune system, thereby influencing circulating levels of cytokines such as interleukin-6 (IL-6) and tumor necrosis factor-α (TNF-α) [33].

The vagus nerve is the principal neural pathway of the gut–brain axis. Sensory signals from the intestine are transmitted via vagal afferent fibers to the nucleus tractus solitarius, which in turn regulates emotion-related brain regions [34]. The vagus nerve plays an irreplaceable role in mediating gut microbiota on the brain. In animal model experiments, vagotomy eliminates the anxiolytic effects of specific probiotics such as Lactobacillus rhamnosus and their regulatory effects on the HPA axis [35]. The vagus nerve, as a critical neural pathway of the gut–brain axis, not only transmits signals from the intestine but also participates in bidirectional regulation of immune and endocrine systems. The integrity of the vagus nerve is essential for maintaining intestinal immune homeostasis and normal central nervous system function; its dysfunction may lead to neuroinflammation and emotional dysregulation [36].

Gut microbiota can also influence anxiety and depression by modulating HPA axis function [37]. HPA axis is the core endocrine system for coping with stress, and its hyperactivation is closely associated with anxiety and depression [38]. In perimenopausal women, declining estrogen levels can lead to HPA axis dysfunction, thereby exacerbating emotional fluctuations. Gut microbiota can regulate HPA axis activity through metabolites such as butyrate, which can inhibit corticotropin-releasing hormone and thereby attenuate the stress response. Furthermore, gut microbiota can regulate serotonin synthesis by influencing Trp metabolic pathways, with serotonin being a key neurotransmitter for regulating emotion and anxiety [39]. The structural and functional foundations of the gut–brain axis provide an important framework for understanding the pathogenesis of perimenopausal anxiety and depression, and also lay a theoretical foundation for nutritional intervention strategies targeting gut microbiota.

### 3.2. Regulation of Neurotransmitter Metabolism by Gut Microbiota

The gut microbiota extends far beyond digestion and absorption; it can directly synthesize or regulate the precursors or final products of various neurotransmitters. Gut microbiota possesses the capability to independently synthesize multiple neurotransmitters and their precursors, including 5-HT, GABA, and dopamine. One study demonstrated that Lactobacillus and Bifidobacterium can directly produce GABA [40]. These bacteria convert glutamate into GABA through their own glutamate decarboxylase, thereby increasing GABA concentration in the intestine [41]. GABA derived from the intestine regulates the transmission of central signals through multiple pathways. Intestine-derived GABA can enter the liver through portal venous circulation for metabolism and can also cross the blood–brain barrier via specific transporters to regulate central GABAergic neurotransmission [42,43]. However, the extent to which gut-derived GABA meaningfully crosses the human blood–brain barrier to regulate central GABAergic neurotransmission directly remains uncertain and heavily debated. Lambiase C et al. suggest that gut- or microbiome-derived GABA can modulate the central nervous system and host behavior through peripheral and indirect pathways within the gut–brain axis. Additionally, another study showed that gut microbiota can also transmit GABA signals from the intestine to the brain through the vagus nerve, thereby influencing emotion and behavior [44]. Escherichia coli, Bacillus, and yeast have also been found to produce dopamine and 5-HT [45]. These microbiota-derived neurotransmitters not only regulate intestinal motility and secretion locally but also affect central nervous system function through circulation or the vagus nerve [46].

Gut microbiota can regulate metabolic flux of neurotransmitter precursors by secreting specific enzymes. The regulation of Trp metabolism by gut microbiota represents a critical component of gut–brain axis function [47]. Trp is the sole precursor for the synthesis of 5-HT and kynurenine (KYN), and the balance of its metabolic pathways is essential for maintaining nervous system homeostasis [48]. Under healthy conditions, the gut microbiota maintains Trp metabolic balance and promotes 5-HT synthesis through its metabolites and immunomodulatory effects [49]. When gut dysbiosis occurs, indoleamine 2,3-dioxygenase (IDO) is upregulated, directing more Trp toward KYN conversion [50]. This shift reduces 5-HT production while increasing KYN accumulation. This metabolic redirection not only decreases the synthesis of neuroprotective 5-HT but also enhances the neurotoxic metabolite quinolinic acid (QA) [51,52,53]. QA is an N-methyl-D-aspartate (NMDA) receptor agonist; its overactivation can induce excitotoxicity and neuronal damage, which is implicated in the pathological processes of depression, schizophrenia, Alzheimer’s disease, and other disorders [54].

### 3.3. Changes in Gut Microbiota During Perimenopause

The significant decline in estrogen levels during perimenopause is a key driving factor for alterations in gut microbiota composition and diversity. These changes ultimately promote anxiety and depression by disrupting intestinal barrier function and triggering systemic low-grade inflammation. Estrogen can reduce intestinal permeability and prevent “leaky gut” by regulating tight junction protein expression through its receptors [55]. The decrease in estrogen levels during perimenopause leads to impaired intestinal barrier function, facilitating the translocation of bacterial endotoxin lipopolysaccharide across the intestinal wall into the bloodstream, thereby initiating systemic low-grade inflammation. This chronic low-grade inflammatory state can affect the central nervous system by activating immune cells and releasing pro-inflammatory cytokines, activating neuroinflammatory pathways, and consequently promoting anxiety and depression [56]. Compared with women of reproductive age, the gut microbiota of perimenopausal women exhibits a trend of decreased beneficial bacteria and increased potential pathogens. The abundance of genera with anti-inflammatory and intestinal barrier-maintaining functions, such as Lactobacillus and Bifidobacterium, is reduced, while the relative abundance of Bacteroidetes and Proteobacteria increases [57]. This dysbiosis is not only closely associated with systemic low-grade inflammation but is also considered an important contributor to perimenopausal anxiety and depression. A study of perimenopausal patients with panic disorder found that their gut microbiota α-diversity was significantly decreased, and Bacteroides and Alistipes were positively correlated with anxiety and panic symptoms [58]. Bacteroides and Alistipes can degrade intestinal Trp, disrupting systemic Trp availability, thereby affecting 5-HT synthesis and leading to depressive-like behaviors. An animal experiment has further confirmed that ovariectomy (OVX)-induced perimenopausal mouse models exhibit obvious depressive-like behaviors, accompanied by enrichment of microbial taxa previously associated with inflammatory phenotypes [59]. Through fecal microbiota transplantation experiments, researchers found that transferring gut microbiota from perimenopausal women or ovariectomized animals into germ-free mice could induce anxiety- and depression-like behavioral phenotypes in recipient mice [60]. Another study demonstrated that transferring fecal microbiota from OVX mice to normal mice induced depressive-like behaviors; conversely, transferring microbiota from young female mice to OVX mice ameliorated their depression [61].

## 4. Trp Metabolism and Perimenopausal Anxiety and Depressions

### 4.1. Trp Metabolic Pathways

Trp is an essential amino acid in humans, and its unbalanced metabolic pathways are connected to anxiety and depression in perimenopausal women [62]. Trp metabolism in host cells is primarily divided into the kynurenine pathway (KP), the serotonin pathway, and the indole pathway. Approximately 95% of Trp is metabolized through the KP, catalyzed by IDO or tryptophan 2,3-dioxygenase (TDO), converting Trp into KYN [63]. Trp can also be metabolized through the serotonin pathway, where tryptophan hydroxylase (TPH) catalyzes its conversion to 5-HT [64]. Additionally, gut microbiota can convert Trp into indole-3-propionic acid and indole-3-aldehyde, which participate in immune regulation by activating the aryl hydrocarbon receptor [65].

### 4.2. Trp with Anxiety and Depression

5-HT is a key neurotransmitter regulating emotion, sleep, and appetite; its insufficient synthesis severely impairs emotional regulatory circuits such as the amygdala and prefrontal cortex [66]. Approximately 90% of 5-HT synthesis occurs in the intestine, with the remaining 10% synthesized in the brain [49]. During perimenopause, fluctuations in estrogen levels can affect Trp metabolic enzyme activity, leading to reduced 5-HT synthesis, which is closely associated with depression [67]. During perimenopause, fluctuations in estrogen levels can affect Trp metabolic enzyme activity, leading to reduced 5-HT synthesis, which is closely associated with the development of depression [68]. Estrogen physiologically inhibits IDO expression. With estrogen decreased, IDO activity increases [69]. As the rate-limiting enzyme for Trp entry into KP, enhanced IDO activity directly reduces Trp availability for 5-HT synthesis, thereby affecting emotional regulation. Estrogen also promotes 5-HT synthesis by upregulating tryptophan hydroxylase-2 (TPH-2) expression and inhibiting monoamine oxidase type A (MAO-A) to reduce 5-HT degradation [70]. When estrogen levels decline, this protective mechanism is compromised, directing Trp metabolism toward the neurotoxic KP.

Perimenopause is frequently accompanied by a low-grade inflammatory state, which further exacerbates Trp metabolic imbalance. As estrogen levels decrease, IL-6 and TNF-α levels rise. These cytokines are potent activators of IDO, further driving Trp metabolism toward the kynurenine pathway [11]. Kynurenine pathway metabolites include kynurenic acid (KYNA) and QA, which maintain a dynamic balance between neuroprotection and neurotoxicity. KYNA serves as an antagonist of N-methyl-D-aspartate receptors (NMDAR), exerting neuroprotective effects [71]. QA is an N-methyl-D-aspartate (NMDA) receptor agonist; its excessive accumulation can induce glutamatergic system overactivation and excitotoxicity, which is closely related to the pathophysiological processes of anxiety and depression [72]. In perimenopausal women, the kynurenine pathway is overactivated with increased QA generation, while neuroprotective KYNA levels are significantly reduced [73]. This metabolic imbalance is not only associated with depression but may also aggravate cognitive decline and anxiety behaviors.

**Figure 1:**
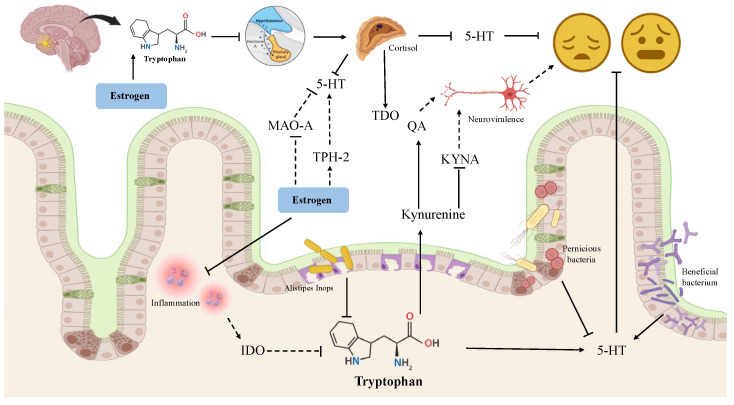
The decline in estrogen levels during perimenopause is a core factor triggering Trp metabolic imbalance. Reduced estrogen weakens the inhibition of IDO and MAO-A, while simultaneously compromising the promotion of TPH-2, leading to decreased 5-HT synthesis. Furthermore, estrogen reduction activates inflammatory responses, driving Trp conversion toward the KYN pathway. Gut microbiota dysbiosis weakens intestinal 5-HT synthesis and exacerbates Trp consumption. Specific bacterial species activate the HPA axis and elevate cortisol levels, inducing enhanced TDO activity and reducing 5-HT, ultimately contributing to perimenopausal depression and anxiety.

Furthermore, gut microbiota is important in Trp metabolism. Perimenopausal women exhibit reduced gut microbiota α-diversity and decreased beneficial bacteria [58]. Gut microbiota is an important source of 5-HT in the body. The reduction in Lactobacillus and other beneficial bacteria directly weakens intestinal 5-HT synthesis capacity [74]. Additionally, abnormal proliferation of specific gut bacteria, such as Alistipes inops, can degrade intestinal Trp, disrupting systemic Trp availability and further exacerbating 5-HT deficiency [61]. Trp metabolic imbalance further leads to HPA axis hyperactivation, which is closely related to neuroendocrine dysregulation caused by estrogen decline. Elevated cortisol not only directly promotes Trp metabolism through the kynurenine pathway but also directly inhibits 5-HT synthesis [75]. As cortisol levels rise in the body, hepatic TDO activity increases. Similar to IDO in function, TDO catalyzes Trp entry into the kynurenine pathway, exacerbating neurotoxicity [76]. The solid lines in the Figure are supported by human evidence, while the dashed lines indicate inferences based on preclinical or mechanistic studies (Figure 1).

## 5. GABA and Perimenopausal Anxiety and Depression

### 5.1. GABA Synthesis and Regulation

GABA is the most important inhibitory neurotransmitter in the central nervous system (CNS), playing a key role in regulating emotion, stress response, and sleep. GABA synthesis primarily depends on GAD, which catalyzes the decarboxylation of glutamate to produce GABA [77]. Synthesized GABA acts on GABA-A and GABA-B receptors to inhibit neuronal overexcitation, thereby maintaining neural network stability [78]. In addition to GABA synthesized by the CNS itself, the gut microbiota is also an important source of GABA in the body. Lactobacillus and Bifidobacterium possess GAD and can utilize intestinal glutamate to synthesize GABA [46]. GABA produced by gut microbiota can stimulate vagal nerve endings on the intestinal wall, transmitting signals to the brainstem and subsequently affecting the amygdala and prefrontal cortex, which are involved in emotional regulation [79]. GABA can also regulate intestinal barrier function. GABA can enhance tight junctions between intestinal epithelial cells, reduce intestinal permeability, and prevent harmful substances in the intestine from entering the bloodstream, thereby maintaining intestinal barrier integrity [80]. This protective effect on the intestinal barrier is crucial for healthy gut–brain axis communication. When intestinal barrier function is impaired, it triggers systemic low-grade inflammatory responses, affecting neurotransmitter metabolism and neuroinflammation [81].

### 5.2. GABA with Anxiety and Depression

Estrogen can regulate GABA synthesis, release, and GABA-A receptor expression and function. The decline in estrogen leads to decreased GABAergic system function, which is associated with a high incidence of anxiety, depression, and insomnia in perimenopausal women [82]. Weakened GABAergic system function reduces the inhibitory capacity over neuronal excitability, making the brain more sensitive to stressors and thereby increasing anxiety and depression [83]. A clinical study found GABA in the medial prefrontal cortex of perimenopausal women was significantly lower than that in women of reproductive age, and was negatively correlated with age, suggesting that decreased brain GABAergic system function is a key factor contributing to increased depression risk in perimenopausal women [84]. When GABAergic system function is compromised, GABA-A receptor dysfunction leads to disinhibition of emotion-processing centers such as the amygdala, causing amygdala overactivation and thereby enhancing fear and anxiety responses in individuals [85]. Perimenopausal women often experience sleep disturbances; decreased GABA levels not only directly cause emotional dysregulation but may also indirectly exacerbate anxiety and depression by disrupting sleep architecture [86]. In the pathogenesis of depression, the functional decline of GABAergic interneurons also plays a key role. Parvalbumin-positive neurons are the main inhibitory interneurons in the brain, responsible for regulating the rhythmic activity of local neural circuits [87]. In perimenopausal women with depression, decreased function of GABAergic interneurons disrupts the excitation/inhibition (E/I) balance in key brain regions [28]. E/I imbalance disrupts emotional regulation networks, resulting in relatively enhanced excitatory glutamatergic transmission and weakened inhibitory GABAergic transmission, thereby triggering or aggravating depression [88].

Furthermore, HPA axis hyperactivation during perimenopause further impairs GABAergic system function. Estrogen fluctuations and withdrawal can damage HPA axis negative feedback regulation mechanisms, leading to persistently elevated cortisol levels [89]. Under chronic stress, high cortisol levels not only directly act on emotion-regulating brain regions but also downregulate GABA-A receptor expression and function, reducing their sensitivity to GABA [90]. This adaptive change at the receptor level results in decreased inhibitory signal transduction efficiency even with adequate GABA release, forming a functionally hypofunctional GABAergic state [91]. Perimenopausal women often experience elevated perceived stress levels, and the vicious cycle between stress and anxiety further exacerbates GABAergic system dysfunction.

Additionally, gut microbiota are important in GABAergic system function during perimenopause. Intestinal Lactobacillus and Bifidobacterium possess GABA-synthesizing capacity and serve as an important source of circulating GABA in the body [92]. Perimenopausal women exhibit reduced gut microbiota diversity, decreased abundance of beneficial bacteria such as Lactobacillus and Bifidobacterium, and relative enrichment of pro-inflammatory bacteria. This alteration in microbiota structure directly reduces intestinal GABA synthesis capacity, lowering GABA levels entering the circulatory system and subsequently affecting central GABAergic system function [93]. Gut microbiota dysbiosis can also indirectly influence GABAergic system function through increased intestinal permeability, immune-inflammatory pathways, and interference with Trp metabolism [94].

**Figure 2:**
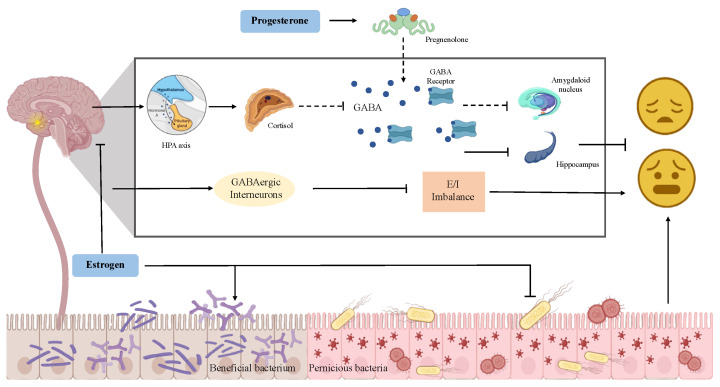
The decline in GABAergic system function during perimenopause is a core factor triggering anxiety and depression. Reduced estrogen directly weakens GABA synthesis, release, and GABA-A receptor function. Decreased progesterone leads to reduced allopregnanolone, attenuating the inhibitory modulation of GABA-A receptors. Together, these changes result in insufficient GABAergic inhibition, overactivation of emotion-processing centers such as the amygdala, and disruption of excitation/inhibition balance in the prefrontal cortex and hippocampus. Concurrently, declining estrogen leads to HPA axis hyperactivation; elevated cortisol levels downregulate GABA-A receptor expression, creating a functionally hypofunctional GABAergic state. Reduced beneficial bacteria decrease intestinal GABA synthesis. Meanwhile, microbiota dysbiosis indirectly impairs GABAergic function through activation of inflammatory pathways and increased intestinal permeability, ultimately contributing to perimenopausal depression and anxiety.

The significant decline in progesterone levels is also an important cause of weakened GABAergic system function. Allopregnanolone, a neuroactive metabolite of progesterone, is a potent positive allosteric modulator of GABA-A receptors, capable of enhancing GABA-mediated inhibitory neurotransmission [95]. Ovarian function declines during perimenopause, leading to reduced progesterone secretion, consequently lowering allopregnanolone levels, directly weakening GABA inhibitory effects, and resulting in relatively increased central nervous system excitability, thereby increasing susceptibility to anxiety and depression [96]. This hormone-driven change constitutes an important physiological basis for the increased risk of anxiety and depression in perimenopausal women. The solid lines in the Figure are supported by human evidence, while the dashed lines indicate inferences based on preclinical or mechanistic studies (Figure 2).

## 6. Potential Nutritional Intervention Strategies

### 6.1. Nutritional Strategies Targeting Trp Metabolism

#### 6.1.1. Trp and 5-HT Precursor Supplementation

Direct Trp supplementation is a straightforward strategy to increase Trp concentration and enhance brain 5-HT synthesis. Trp-enriched diets or supplements have been shown to effectively improve social cognitive function [97]. In a double-blind study, 4 weeks of a Trp-enriched diet and acute 5-hydroxytryptophan (5-HTP) intake altered neural activity related to emotion recognition [98]. Additionally, Trp can directly interact with tubulin to promote microtubule assembly, influencing neuroplasticity and memory storage, thereby alleviating brain fog symptoms and reducing anxiety and depression in perimenopausal women [99]. Although Trp supplementation can significantly reduce the occurrence of negative emotions, this strategy has certain limitations. First, approximately 95% of ingested Trp is metabolized through the KYN pathway, with only a small fraction utilized for 5-HT synthesis. Second, Trp competes with other large neutral amino acids in plasma, which restricts its transport across the blood–brain barrier. Therefore, co-ingestion with carbohydrates is crucial. Insulin promotes muscle uptake of branched-chain amino acids, thereby reducing plasma branched-chain amino acid levels and relatively increasing Trp’s competitive advantage to enhance its blood–brain barrier permeability [100].

As the direct precursor of 5-HT, 5-HTP supplementation can more efficiently elevate 5-HT levels. In a randomized double-blind placebo-controlled crossover trial in Parkinson’s disease patients, 50 mg daily 5-HTP for 4 weeks significantly improved Hamilton Depression Rating Scale scores [101]. Another randomized controlled trial in elderly Singaporeans also confirmed that 100 mg daily 5-HTP for 12 weeks increased 5-HT, improved Montreal Cognitive Assessment scores, and reduced Geriatric Depression Scale scores, thereby enhancing cognitive function and alleviating depression [102]. The timing of 5-HTP supplementation warrants attention; postprandial or bedtime administration is recommended, as carbohydrate intake at these times promotes insulin secretion, helping to increase the Trp/LNAA ratio and enhance 5-HTP brain entry efficiency. However, the safety of the 5-HTP dosage and the potential conflicts when taken together with other medications must be strictly controlled; otherwise, serious adverse reactions may occur. A case report described a 44-year-old male who accidentally ingested 10 times the recommended dose of 5-HTP powder and developed severe reversible hippocampal ischemia, manifesting as anterograde and retrograde amnesia, disorientation, and confusion [103]. 5-HTP may also interact with some serotonin-based drugs. In a rat model, it was observed that when the non-selective MAO inhibitor pargyline and the selective MAO-A inhibitor clorgyline were administered together with 5-HTP, the rats exhibited convulsions and an increase in body temperature [104].

#### 6.1.2. Inhibition of KP

The excessive diversion of Trp toward the kynurenine pathway is considered one of the key pathological mechanisms underlying anxiety and depression in perimenopausal women. This process is primarily catalyzed by IDO and TDO, whose activities are significantly upregulated by inflammatory factors and stress states. Inhibiting IDO or TDO activity represents a critical strategy to reduce Trp diversion toward the kynurenine pathway and increase availability for 5-HT synthesis. Currently, several natural compounds have demonstrated potential IDO inhibitory activity [105]. Curcumin has been confirmed to significantly suppress IFN-γ-induced IDO expression and activity in dendritic cells by blocking the JAK-PKCδ-STAT1 signaling pathway [106]. Similar to curcumin, resveratrol can also inhibit IDO transcription and functional activity through JAK/STAT1 and PKC-δ-dependent signaling pathways, showing inhibitory effects on IDO1 enzyme activity [107]. Quercetin attenuates IDO/TDO activity through antioxidant and anti-inflammatory mechanisms, effectively reducing the production of inflammatory factors such as IFN-γ and IL-6 [108].

Beyond inhibiting upstream diversion, interventions targeting downstream metabolites of the kynurenine pathway also hold potential. The kynurenine pathway does not exclusively produce harmful substances; its metabolite KYNA is an N-methyl-D-aspartate (NMDA) receptor antagonist with neuroprotective properties. Significantly reduced KYNA in the prefrontal cortex has been observed in genetic rat models of depression, with KYNA reduction associated with depressive pathology [109]. Studies have shown that administration of the KYNA analogue 7-chlorokynurenic acid can activate hippocampal BDNF signaling through the TrkB-ERK/Akt pathway, producing rapid antidepressant-like effects [110,111].

Vitamin B6, as an essential cofactor for multiple key enzymes in the kynurenine pathway, plays an indispensable role in regulating Trp metabolic balance [112]. Vitamin B6 serves not only as a coenzyme for kynureninase and 3-hydroxykynureninase, catalyzing the conversion of KYN to anthranilic acid and 3-hydroxykynurenine to 3-hydroxyanthranilic acid, but also as a necessary cofactor for kynurenine aminotransferase in synthesizing neuroprotective KYNA [113]. Vitamin B6 directly participates in the synthesis of neurotransmitters, and it is crucial for maintaining normal neurological function [114]. Vitamin B6 deficiency leads to accumulation of neurotoxic metabolites such as 3-hydroxykynurenine and xanthurenic acid, while simultaneously reducing KYNA and anthranilic acid production, disrupting metabolic balance [115]. Perimenopausal women often face increased risk of B-vitamin deficiency due to dietary changes, decreased absorption capacity, or increased metabolic demands. Rational vitamin B6 supplementation can optimize enzyme activity in the kynurenine pathway, promote metabolic flux toward the neuroprotective KYNA branch, and concurrently reduce QA generation, thereby exerting positive effects on mood.

#### 6.1.3. Modulation of Gut Microbiota to Increase Trp

Gut microbiota dysbiosis in perimenopausal women is closely associated with the development of mood disorders. One core mechanism involves significantly reduced gut microbial diversity, decreased SCFA-producing bacteria, and increased pro-inflammatory bacteria [116]. Alterations in gut microbiota structure interfere with the Trp-KYN metabolic pathway by affecting intestinal barrier function, immune activation, and HPA axis activity [117].

Probiotic supplementation is a direct approach to modulate gut microbiota and enhance intestinal 5-HT synthesis capacity [36]. In perimenopausal women, supplementation with probiotics helps restore beneficial bacteria depleted by estrogen fluctuations and improves gut microecological balance [116]. Lactiplantibacillus plantarum DR7 can upregulate intestinal tryptophan hydroxylase-2 (TPH2) expression while downregulating IDO and TDO activity, thereby promoting Trp conversion toward 5-HT synthesis and reducing diversion to the KP [118]. A randomized double-blind study demonstrated a 12-week DR7 intervention significantly reduced anxiety (*p* = 0.001) and stress (*p* = 0.024) levels in stressed adults, concurrently decreasing plasma cortisol and pro-inflammatory factors while increasing the anti-inflammatory factor IL-10 [119]. Additionally, probiotics can indirectly alleviate negative impacts on Trp metabolism by enhancing intestinal barrier function, reducing endotoxin translocation into blood, and suppressing systemic low-grade inflammation [120].

Prebiotics such as fructooligosaccharides and inulin indirectly regulate Trp metabolism through selectively promoting beneficial bacterial proliferation [121]. Dietary fiber fermented by gut microbiota produces SCFAs, particularly butyrate, which exerts important immunomodulatory effects [122]. Butyrate can inhibit histone deacetylases, exerting anti-inflammatory and neuroprotective effects [123]. Butyrate can reduce excessive Trp conversion toward the KP by modulating intestinal barrier function and neuroimmune pathways, thereby preserving more Trp for 5-HT synthesis [124]. The fiber- and polyphenol-rich Mediterranean dietary pattern has been confirmed to increase SCFA-producing bacterial abundance through polyphenols, fiber, and ω-3 fatty acids, while alleviating inflammation and neuroendocrine stress responses, thereby relieving mood disorders [125,126].

The detailed description of different nutritional strategies for regulating tryptophan metabolism is presented in Table 1.

### 6.2. Nutritional Strategies Targeting GABA Metabolism

#### 6.2.1. GABA Supplementation and Precursor Intervention

Oral GABA supplements have gained attention as dietary supplements for alleviating anxiety and improving sleep in perimenopausal women. However, their limited efficiency in crossing the blood–brain barrier restricts direct action on the central nervous system. A systematic review noted that oral GABA (dose range of 20–300 mg/day) could reduce stress marker chromogranin A (CgA) and cortisol levels, and improve heart rate variability (HRV) [127].

Furthermore, glutamine serves as a precursor for GABA synthesis and plays an important role in emotional regulation [128]. Glutamine is not only an important energy substrate for intestinal mucosal cells and immune cells, but also a key component of the glutamate–glutamine cycle in the brain. Supplementation with 450 μg/25 g mouse/day (equivalent to 90 mg/day in humans) for 3 weeks to 4 months significantly reduced plasma corticosterone levels in mice, alleviated despair behaviors, and activated glutamatergic neurotransmission [129]. In human studies, higher doses of glutamine (40 g/day) improved mood states in bone marrow transplant patients [130].

Taurine, as an agonist modulator of GABA-A and glycine receptors, possesses clear neuroprotective and antidepressant potential. Electrophysiological studies have confirmed that taurine at physiological concentrations (10–100 μM) is a potent activator of extrasynaptic GABA-A receptors in thalamic VB neurons, effectively exerting neuromodulatory and neuroprotective effects [131]. Animal model studies have shown that taurine levels in the medial prefrontal cortex (mPFC) are significantly reduced in mice with depression induced by chronic social defeat stress. Taurine supplementation protects cortical neuronal dendritic spine density. It restores NMDA receptor NR2A subunit expression, effectively improving depressive-like behaviors [132].

#### 6.2.2. Enhancing Endogenous GABA Synthesis

As the primary inhibitory neurotransmitter in the brain, promoting endogenous GABA synthesis through nutritional intervention represents a promising strategy for improving emotional states in perimenopausal women. Vitamin B6 is a critical cofactor for glutamate decarboxylase (GAD), the rate-limiting enzyme catalyzing glutamate conversion to GABA [133]. Perimenopausal women often experience decreased vitamin B6 levels due to unbalanced dietary patterns, metabolic alterations, or hormonal fluctuations, which may consequently impair GABA synthesis efficiency. A randomized controlled trial for premenstrual syndrome (PMS) demonstrated that vitamin B6 treatment over three menstrual cycles produced significantly superior overall symptom improvement compared to placebo (*p* < 0.02) [134]. A study in young adults found that high-dose vitamin B6 supplementation (100 mg/day) significantly reduced anxiety levels, with mechanisms related to enhanced central GABA production [135].

Magnesium ions serve as positive allosteric modulators of GABA-A receptors, enhancing GABA binding affinity to its receptors and thereby amplifying GABA inhibitory signals. Magnesium deficiency further activates the HPA axis and elevates cortisol, which has been confirmed to be associated with anxiety and depression [136]. A study showed that magnesium supplementation significantly reduced depression scores (SMD = −0.919, *p* = 0.001), though high heterogeneity among studies limited evidence quality [137]. Another systematic review indicated that magnesium supplementation improved subjective anxiety, but did not support its use as monotherapy [138]. For perimenopausal women, magnesium supplementation may help improve sleep quality and indirectly support emotional stability through enhanced GABAergic inhibition; however, direct anxiolytic effects remain unestablished.

L-theanine, a unique amino acid present in green tea, also demonstrates significant capacity to promote GABA synthesis and release. L-theanine can cross the blood–brain barrier, increasing brain levels of GABA, dopamine, 5-HT, and other neurotransmitters, thereby exerting relaxation, anxiety relief, and sleep improvement effects [139]. A meta-analysis encompassing 19 RCTs demonstrated that L-theanine (200–400 mg/day) significantly improved subjective sleep quality (SMD = 0.43, *p* = 0.03), shortened sleep latency (SMD = 0.15, *p* = 0.04), and reduced daytime dysfunction (SMD = 0.33, *p* < 0.001) [140].

#### 6.2.3. Modulation of Gut Microbiota to Increase GABA

Specific strains of GABA-producing probiotics, including *Lactobacillus brevis* and *Bifidobacterium dentium*, have demonstrated GABA-producing capacity only in vitro and in animal models to date. In vitro studies show that *L. brevis* harbors two GAD-encoding genes, gadA and gadB. gadB forms a gadCB operon with upstream gadC, and its expression is significantly upregulated under acid stress conditions, substantially increasing GABA yield [141]. Genomic analysis further confirmed that *L. brevis* CRL 2013 contains no antibiotic resistance genes or virulence markers, with GABA production activated by the gadR transcriptional regulator, achieving functional-level GABA output under optimized conditions [142]. However, these mechanistic findings cannot be extrapolated to clinical efficacy in humans. *B. dentium* produces GABA in the intestine through a gadB-dependent mechanism. Animal experiments demonstrated that oral administration of wild-type *B. dentium* significantly reduced nociceptive responses in a rat visceral hypersensitivity model, confirming that microbiota-derived GABA can regulate peripheral nerve activity through the gut–brain axis [143]. Furthermore, mono-colonization with *B. dentium* significantly altered GABA, glutamate, and glutamine concentrations in both the intestines and brains of mice, indicating that gut microbiota-derived GABA can systemically influence host neurotransmitter balance [144]. These findings are strain-specific and species-dependent. However, no clinical studies have yet confirmed that oral administration of these strains can directly increase human serum GABA concentrations or improve perimenopausal emotional symptoms; their clinical translation still requires validation through rigorously designed randomized controlled trials.

Traditional fermented foods are rich in lactic acid bacteria capable of producing GABA, and regular consumption of these foods helps maintain metabolic diversity of the gut microbiota and improve mood disorders [145]. By screening high GABA-yielding probiotic combinations such as *L. lactis*, *L. kefiri*, and *L. acidophilus*, fermented whey beverages with functional-level GABA content can be prepared [146]. An animal study found the regulatory effects of Lactiplantibacillus plantarum LPB145, a strain isolated from Uruguayan artisanal cheese starter with potential probiotic characteristics and GABA-producing capability, on anxiety and depression behaviors [43]. Plant-based kefir beverages have also been confirmed as natural sources of GABA, possessing antioxidant and antidepressant potential [147]. For perimenopausal women, increasing fermented food intake may potentially improve mood disorders by enhancing gut microecological balance.

The prebiotic galactooligosaccharide can selectively promote proliferation of GABA-producing strains such as *L. brevis* and *B. dentium*, thereby enhancing intestinal GABA synthesis efficiency [148]. GOS increased Bifidobacterium abundance and produced acetic acid and propionic acid in an in vitro fermentation model. These metabolites can modulate the intestinal environment and influence GABA production [149].

The detailed description of different nutritional strategies for regulating GABA is presented in Table 2.

## 7. Discussion

Perimenopause represents a critical physiological transitional stage in women’s lives, with the high incidence of anxiety and depression becoming a significant public health issue affecting this population’s quality of life. Nutritional intervention represents a promising but still investigational non-pharmacological approach that provides potential avenues for supporting the management of perimenopausal anxiety and depression. Gut microbiome composition is influenced by numerous confounding factors, including diet, age, BMI, medications, antibiotics, geography, and host genetics. Taxonomic changes do not necessarily reflect functional metabolic alterations because microbial function is strain-specific and metabolite-dependent. Causal relationships between microbiome alterations, Trp metabolism, GABA metabolism, and mood disorders remain incompletely understood. Therefore, future longitudinal multi-omics studies and well-designed randomized controlled trials are needed. Most intervention studies enrolled relatively small cohorts and were underpowered to detect clinically meaningful differences. Most probiotic interventions lasted only 4–12 weeks, making it difficult to determine whether microbiome alterations and psychological improvements are sustained over time. Effective dosages, optimal supplementation timing, and long-term safety of natural nutrients require further validation. Furthermore, it is worth noting regarding the supplementation of probiotics that the effects of probiotics are species-specific, dose-specific, dependent on viability, and have characteristics specific to certain strains. It cannot be concluded that all Lactobacillus or Bifidobacterium strains can improve anxiety, depression, tryptophan metabolism, or GABA production. Changes in gut microbial composition following probiotic supplementation may be transient. Probiotic-associated microbiome alterations often diminish after discontinuation of supplementation, and persistent colonization is uncommon [150]. Therefore, improvements in microbial composition may require sustained dietary or lifestyle interventions rather than short-term probiotic administration. Regarding the safety issues of supplements, such as possible adverse reactions, drug–drug or drug–food interactions, and changes in dosage, more in vitro and in vivo studies are needed to confirm these aspects. For instance, 5-HTP and tryptophan have potential lethal interactions with SSRIs, SNRIs, MAOIs, triptans, and other serotonergic agents; GABA products have uncertain CNS penetration and variable supplement quality; and curcumin/resveratrol/quercetin supplements have known bioavailability issues, GI side effects, interactions with anticoagulants or antiplatelets, hepatotoxicity concerns reported for some preparations, etc. Therefore, nutritional supplements should not be considered inherently safe, and individualized assessment is recommended, particularly in women receiving pharmacological treatment.

## 8. Conclusions and Perspectives

This review addressed perimenopausal mood disorders by elaborating their pathogenesis from the perspectives of epidemiological characteristics, hormonal changes, gut–brain axis function, Trp metabolic disturbance, and GABAergic system hypofunction. The review particularly focused on Trp and GABA metabolic regulation within the gut–brain axis, exploring close associations between perimenopausal mood disorders and these two systems. Fluctuating estrogen levels during perimenopause lead to gut microbiota dysbiosis, impaired intestinal barrier function, and the activation of systemic low-grade inflammation. Gut microbiota disturbance affects Trp metabolism, directing more Trp conversion through the kynurenine pathway toward neurotoxic QA, resulting in decreased 5-HT levels and triggering depression and anxiety. Concurrently, reduced GABA-producing bacteria, decreased endogenous GABA synthesis, and downregulated GABA-A receptor function collectively weaken GABAergic inhibitory capacity, causing overactivation of emotion-processing centers such as the amygdala and disrupting excitation/inhibition balance in the prefrontal cortex and hippocampus, thereby exacerbating anxiety and depression. The potential nutritional intervention strategies summarized in this review are primarily categorized into Trp metabolism-targeted interventions and GABA metabolism-targeted interventions. These strategies can modulate Trp and GABA metabolism, improve gut–brain axis function, and alleviate anxiety and depression in perimenopausal women. Future research should prioritize conducting randomized controlled trials for perimenopausal mood disorders to verify the efficacy and safety of nutritional interventions. Through multidisciplinary collaboration translating basic research findings into clinical practice, additional evidence-based nutritional approaches for adjunctive support in managing perimenopausal mood disorders may emerge, ultimately improving the physical and mental health and quality of life for this population.

## Figures and Tables

**Table 1:** Different nutritional strategies regulate tryptophan metabolism.

Supplement	Subject	Type	Pathway	Mechanisms	Effect	Ref.
Tryptophan	Humans Animals	In vivo (randomized controlled trials, animal experiments)	Direct addition	Increase tryptophan Enhance 5-HT in brain Promote assembly of microtubules	Relieve cognitive function Improve neural activity Alleviate brain fog Reduce anxiety Reduce depression	[97,98,99,100]
5-HTP	Humans	In vivo (randomized controlled trials)	Precursor supplementation	Increase 5-HT Increase the ratio of Trp/LNAA	Improve depression Enhance cognition	[101,102,103,104]
Curcumin	Cells	Ex vivo (cell experiments)	IDO inhibitors	Block JAK-PKCδ-STAT1 Inhibit IDO	Reduce diversion of tryptophan to the kynurenine pathway	[106]
Resveratrol	Cells	Ex vivo (cell experiments)				[107]
Quercetin	Animals	In vivo (animal experiment)		Anti-inflammatory Antioxidant Inhibit IDO		[108]
7-Cl-KYNA	Animals	In vivo (animal experiment)	Downstream metabolite intervention	KYNA analogues	Anti-depression	[109,110,111]
Vitamin B6	Humans Animals	In vivo	Regulate enzyme activity	Reduce quinolinic acid Increase 5-HT and GABA	Reduce neurotoxic metabolites Optimize tryptophan metabolism	[112,113,114,115]
Probiotics	Humans Animals	In vivo (randomized, double-blind, placebo-controlled trial, animal experiment)	Regulate intestinal microbiota	Increase TPH2 Decrease IDO and TDO Regulate intestinal barrier Inhibit inflammation	Reduce inflammation	[116,117,118,119,120]
Prebiotics	Humans Animals	In vivo		Promote beneficial bacteria Anti-inflammatory Regulate intestinal barrier	Increase 5-HT synthesis	[112,113,114,115]
Mediterranean diet pattern	Humans Animals	In vivo		Increase SCFA-producing bacteria Alleviate inflammation Reduce stress responses	Improve intestinal flora Relieve anxiety Relieve depression	[121,122,123,124,125,126]

**Table 2:** Different nutritional strategies regulate GABA.

Supplement	Subject	Type	Pathway	Mechanisms	Effect	Ref.
GABA	Humans	In vivo (clinical trials)	Direct addition	Increase GABA	Reduce CgA and cortisol Improve HRV	[127]
Glutamine	Humans Animals	In vivo	GABA precursor	Increase GABA synthesis	Reduce plasma corticosterone Activate glutamatergic neurotransmission	[128,129,130]
Taurine	Humans Animals	In vitro (Brain slice electrophysiological experiment) In vivo (animal experiment)	GABA-A and glycine receptor agonists regulators	Activate GABA-A receptor Protect cortical neurons Restore NR2A subunit of NMDA receptor	Improve neuroprotection Reduce depression	[131,132]
Vitamin B6	Humans Animals	In vivo In vitro	Increase endogenous GABA	Catalyzing conversion of glutamate into GABA	Reduce anxiety Enhance central GABA	[133,134,135]
Magnesium	Humans Animals	In vivo	GABA-A receptor positive allosteric modulators	Promote GABA Inhibit HPA axis	Reduce depression Reduce anxiety Improve sleep quality	[136,137,138]
L-Theanine	Humans	In vivo (clinical trials)	Promote synthesis and release of GABA	Increase GABA, dopamine and 5-HT in the brain		[139,140]
Probiotics	Microorganism Animals	In vivo In vitro	Regulate intestinal microbiota	Promote the proliferation of GABA-producing strains	Regulate GABA, glutamate and glutamine	[141,142,143,144]
Prebiotics	Animals	In vivo			Enhance GABA synthesis	[148,149]
Traditional fermented food	Microorganism	In vitro		Increase GABA-producing lactic acid bacteria	Provide GABA natural sources Reduce depression Reduce anxiety	[145,146,147]

## Data Availability

No data were used for the research described in this review.

## References

[1] Meeta M., Digumarti L., Agarwal N., Vaze N., Shah R., Malik S. (2020). Clinical Practice Guidelines on Menopause: An Executive Summary and Recommendations: Indian Menopause Society 2019–2020. J. Mid-Life Health.

[2] Burger H.G., Dudley E.C., Hopper J.L., Shelley J.M., Green A., Smith A., Dennerstein L., Morse C. (1995). The endocrinology of the menopausal transition: A cross-sectional study of a population-based sample. J. Clin. Endocrinol. Metab..

[3] Spicer J., Malaspina D., Blank S.V., Goosens K.A. (2025). Follicle-stimulating hormone: More than a marker for menopause: FSH as a frontier for women’s mental health. Psychiatry Res..

[4] El Khoudary S.R., Greendale G., Crawford S.L., Avis N.E., Brooks M.M., Thurston R.C., Karvonen-Gutierrez C., Waetjen L.E., Matthews K. (2019). The menopause transition and women’s health at midlife: A progress report from the Study of Women’s Health Across the Nation (SWAN). Menopause.

[5] Freeman E.W., Sammel M.D., Lin H. (2009). Temporal associations of hot flashes and depression in the transition to menopause. Menopause.

[6] Ng Q.X., Lim D.Y., Chee K.T. (2020). Reimagining the spectrum of affective disorders. Bipolar Disord..

[7] Balasubramanian I., Abhijita B., Krishnamoorthy Y., Gnanadhas J., Beg M.J., Menon V. (2026). Prevalence and incidence of depressive, anxiety, and insomnia symptoms in perimenopausal and postmenopausal women: Systematic review and meta-analysis. Gen. Hosp. Psychiatry.

[8] Page C.E., Soreth B., Metcalf C.A., Johnson R.L., Duffy K.A., Sammel M.D., Loughead J., Epperson C.N. (2023). Natural vs. surgical postmenopause and psychological symptoms confound the effect of menopause on executive functioning domains of cognitive experience. Maturitas.

[9] Herson M., Kulkarni J. (2022). Hormonal Agents for the Treatment of Depression Associated with the Menopause. Drugs Aging.

[10] National Institute for Health and Care Excellence (NICE) (2019). 2019 Surveillance of Menopause: Diagnosis and Management (NICE Guideline NG23).

[11] Marano G., D’Abate C., Ianes I., Sorrenti G., Traversi G., Esposito R., Pavese F., D’Angelo T., Fuso P., Franceschini G. (2026). The Gut Microbiota in Perimenopausal Anxiety: A Novel Therapeutic Pathway Through Diet. Nutrients.

[12] Jones L.A., Sun E.W., Martin A.M., Keating D.J. (2020). The ever-changing roles of serotonin. Int. J. Biochem. Cell Biol..

[13] Li P., Yang L., Shao X., Zou Z., Shi H., Sun Y., Wu X., Li Z., Li Y., Li Z. (2025). Lactobacillales derived from traditional Xizang dairy products improve insomnia and restore neurotransmitter-metabolic profiles via gut microbiota in PCPA-induced mice. Microbiol. Res..

[14] Lambiase C., Rettura F., Sciumè G.D., Tedeschi R., Grosso A., Cancelli L., Bottari A., Fornai M., Antonioli L., de Bortoli N. (2025). Targeting γ-aminobutyric acid pathways in irritable bowel syndrome: Bridging central nervous system, enteric dysfunction, and the microbiota-gut-brain axis. Front. Pharmacol..

[15] Lambiase C., Cancelli L., Tedeschi R., Grosso A., Rettura F., Salemmo R., Bottari A., Filippini F., Salvadori S., Valdiserra G. (2026). Effects of γ-Aminobutyric Acid (GABA) Supplementation on Symptoms, Quality of Life, Intestinal Permeability, Systemic Inflammation and Gut Microbiota in Patients with IBS-D: A Randomized, Double Blind, Placebo-Controlled, Crossover Pilot Study. Nutrients.

[16] Aslan B., Önal Ö. (2025). Prevalence of depressive symptoms during the menopausal transition in Türkiye: Impact of symptom severity, aging anxiety and health-related quality of life. Climacteric J. Int. Menopause Soc..

[17] Kandasamy G., Almaghaslah D., Almanasef M. (2024). A study on anxiety and depression symptoms among menopausal women: A web based cross sectional survey. Front. Public Health.

[18] Akhmedova A.A., Gorobets L.N. (2024). Features of the clinical picture of affective disorders in women during the menopausal transition and early postmenopause. Zhurnal Nevrol. I Psikhiatrii Im. S. S. Korsakova.

[19] Zhang T., Wan Y., Geng L. (2024). Unraveling the core and bridge menopausal symptoms of perimenopausal women: A network analysis. Menopause.

[20] Chalise G.D., Shrestha S., Thapa S., Bharati M., Pradhan S., Adhikari B. (2022). Health Problems experienced by Peri-menopausal Women and their Perception towards Menopause. J. Nepal Health Res. Counc..

[21] Kang J.H., Kim M.J. (2022). Factors influencing the health-related quality of life in Korean menopausal women: A cross-sectional study based on the theory of unpleasant symptoms. Korean J. Women Health Nurs..

[22] Wang Q., Zhao D., Zhou M., Zhao X., Gao Y., Duan J., Cao C., Li P. (2022). The Effect of Resilience and Family Support Match on Psychological Distress among Women in the Menopausal Transition Based on Polynomial Regression and Response Surface Analysis. Int. J. Environ. Res. Public Health.

[23] Courtney A., Owens L. (2025). Current evidence and research gaps in menopause management in women with type 1 diabetes mellitus: A narrative review. Endocr. Connect..

[24] Gao M., Zhang H., Gao Z., Sun Y., Wang J., Wei F., Gao D. (2022). Global hotspots and prospects of perimenopausal depression: A bibliometric analysis via CiteSpace. Front. Psychiatry.

[25] Gordon J.L., Sander B., Eisenlohr-Moul T.A., Sykes Tottenham L. (2021). Mood sensitivity to estradiol predicts depressive symptoms in the menopause transition. Psychol. Med..

[26] Lozza-Fiacco S., Gordon J.L., Andersen E.H., Kozik R.G., Neely O., Schiller C., Munoz M., Rubinow D.R., Girdler S.S. (2022). Baseline anxiety-sensitivity to estradiol fluctuations predicts anxiety symptom response to transdermal estradiol treatment in perimenopausal women-A randomized clinical trial. Psychoneuroendocrinology.

[27] Perich T., Ussher J. (2021). Stress predicts depression symptoms for women living with bipolar disorder during the menopause transition. Menopause.

[28] Fidecicchi T., Giannini A., Chedraui P., Luisi S., Battipaglia C., Genazzani A.R., Genazzani A.D., Simoncini T. (2024). Neuroendocrine mechanisms of mood disorders during menopause transition: A narrative review and future perspectives. Maturitas.

[29] Sander B., Muftah A., Sykes Tottenham L., Grummisch J.A., Gordon J.L. (2021). Testosterone and depressive symptoms during the late menopause transition. Biol. Sex. Differ..

[30] Wang I.C., Buffington S.A., Salas R. (2024). Microbiota-Gut-Brain Axis in Psychiatry: Focus on Depressive Disorders. Curr. Epidemiol. Rep..

[31] Zeng L., Zhang S., Liu R., Wang L., Tan Y. (2025). The Microbiota-Gut-Brain Axis in Depression: Mechanisms, Microbiota-Targeted Interventions, and Translational Challenges. Int. J. Microbiol..

[32] Akif A., Islam M.R. (2026). The Microbiota-Gut-Brain Axis in the Pathophysiology of Major Depressive Disorder: A Mechanistic Review. Compr. Physiol..

[33] Zhang Z., Liu Y., Zhao B., Fu H., Wang J., Zhang M., Zhang Y., Hu L., Zhang X. (2026). Zhichi Suanzaoren Decoction alleviates perimenopausal insomnia via restoring astrocytic primary cilia and modulating Wnt/GSK-3β/GR signaling axis. J. Ethnopharmacol..

[34] Longo S., Rizza S., Federici M. (2023). Microbiota-gut-brain axis: Relationships among the vagus nerve, gut microbiota, obesity, and diabetes. Acta Diabetol..

[35] Liu Y., Sanderson D., Mian M.F., McVey Neufeld K.A., Forsythe P. (2021). Loss of vagal integrity disrupts immune components of the microbiota-gut-brain axis and inhibits the effect of Lactobacillus rhamnosus on behavior and the corticosterone stress response. Neuropharmacology.

[36] Wang H., Shi F., Zheng L., Zhou W., Mi B., Wu S., Feng X. (2025). Gut microbiota has the potential to improve health of menopausal women by regulating estrogen. Front. Endocrinol..

[37] Goel A., Das S., Mazumder A., Sinha A. (2026). Microbiota-Gut-Brain Axis: A Novel Paradigm in the Neurobiology of Anxiety. CNS Neurol. Disord. Drug Targets.

[38] Yu Z., Feng C., Chen Y., Wang W., Zhao X. (2025). Untargeted metabolomics revealed that quercetin improved adrenal gland metabolism disorders and modulated the HPA axis in perimenopausal depression model rats. J. Steroid Biochem. Mol. Biol..

[39] Ren Q., He C., Sun Y., Gao X., Zhou Y., Qin T., Zhang Z., Wang X., Wang J., Wei S. (2024). Asiaticoside improves depressive-like behavior in mice with chronic unpredictable mild stress through modulation of the gut microbiota. Front. Pharmacol..

[40] Tamés H., Sabater C., Royo F., Margolles A., Falcón J.M., Ruas-Madiedo P., Ruiz L. (2024). Mouse intestinal microbiome modulation by oral administration of a GABA-producing Bifidobacterium adolescentis strain. Microbiol. Spectr..

[41] Ma S.R., Yu J.B., Fu J., Pan L.B., Yu H., Han P., Zhang Z.W., Peng R., Xu H., Wang Y. (2021). Determination and Application of Nineteen Monoamines in the Gut Microbiota Targeting Phenylalanine, Tryptophan, and Glutamic Acid Metabolic Pathways. Molecules.

[42] Koppula S., Wankhede N., Kyada A., Ballal S., Arya R., Singh A.K., Gulati M., Sute A., Sarode S., Polshettiwar S. (2025). The gut-brain axis: Unveiling the impact of xenobiotics on neurological health and disorders. Prog. Neuro-Psychopharmacol. Biol. Psychiatry.

[43] Han M., Dong Y., Wang S., Huang X., Bai C., Gai Z. (2024). Regulation of gut microbiota and serum neurotransmitters in mice by Streptococcus thermophilus GA8- and Lacticaseibacillus rhamnosus HAO9-fermented milk containing high levels of gamma-aminobutyric acid. J. Sci. Food Agric..

[44] Dicks L.M.T. (2022). Gut Bacteria and Neurotransmitters. Microorganisms.

[45] Dicks L.M.T. (2023). Our Mental Health Is Determined by an Intrinsic Interplay between the Central Nervous System, Enteric Nerves, and Gut Microbiota. Int. J. Mol. Sci..

[46] Qu S., Yu Z., Zhou Y., Wang S., Jia M., Chen T., Zhang X. (2024). Gut microbiota modulates neurotransmitter and gut-brain signaling. Microbiol. Res..

[47] Zhang Q., Zhao W., Yun Y., Ma T., An H., Fan N., Wang J., Wang Z., Yang F. (2025). Multiomics analysis reveals aberrant tryptophan-kynurenine metabolism and immunity linked gut microbiota with cognitive impairment in major depressive disorder. J. Affect. Disord..

[48] Holeček M. (2026). Serotonin, Kynurenine, and Indole Pathways of Tryptophan Metabolism in Humans in Health and Disease. Nutrients.

[49] Liaqat H., Parveen A., Kim S.Y. (2022). Neuroprotective Natural Products’ Regulatory Effects on Depression via Gut-Brain Axis Targeting Tryptophan. Nutrients.

[50] Roth W., Zadeh K., Vekariya R., Ge Y., Mohamadzadeh M. (2021). Tryptophan Metabolism and Gut-Brain Homeostasis. Int. J. Mol. Sci..

[51] Cheng S., Zhu Z., Li H., Wang W., Jiang Z., Pan F., Liu D., Ho R.C.M., Ho C.S.H. (2023). Rifaximin ameliorates depression-like behaviour in chronic unpredictable mild stress rats by regulating intestinal microbiota and hippocampal tryptophan metabolism. J. Affect. Disord..

[52] Zhou M., Fan Y., Xu L., Yu Z., Wang S., Xu H., Zhang J., Zhang L., Liu W., Wu L. (2023). Microbiome and tryptophan metabolomics analysis in adolescent depression: Roles of the gut microbiota in the regulation of tryptophan-derived neurotransmitters and behaviors in human and mice. Microbiome.

[53] Sun P., Wang M., Li Z., Wei J., Liu F., Zheng W., Zhu X., Chai X., Zhao S. (2022). Eucommiae cortex polysaccharides mitigate obesogenic diet-induced cognitive and social dysfunction via modulation of gut microbiota and tryptophan metabolism. Theranostics.

[54] Kearns R. (2024). Gut-Brain Axis and Neuroinflammation: The Role of Gut Permeability and the Kynurenine Pathway in Neurological Disorders. Cell. Mol. Neurobiol..

[55] Li Z., Zheng Y., Shen F., Zhou X. (2026). Sex hormones and functional gastrointestinal disorders in menopausal women. Front. Endocrinol..

[56] Yu X., Zuo Y., Yang Y., Cheng W., Shi M., Cheng L., Shao Q., Xu Y., Chen L. (2025). Mechanism of Microbiota-Gut-Brain in Perimenopausal Depression: An Inflammatory Perspective. Expert Rev. Mol. Med..

[57] Wang X., Lin Y., Zhou M. (2026). Pathogenesis and potential therapies for perimenopausal depression: Insights from the estrogen-gut microbiota axis. Front. Neuroendocrinol..

[58] Lin S., Wang H., Qiu J., Li M., Gao E., Wu X., Xu Y., Chen G. (2023). Altered gut microbiota profile in patients with perimenopausal panic disorder. Front. Psychiatry.

[59] Lei H., Liu J., Deng J., Zou P., Zou Z., Li Z., Li H., Luo L., Tan Z. (2024). Behavior, hormone, and gut microbiota change by YYNS intervention in an OVX mouse model. Front. Cell. Infect. Microbiol..

[60] Yang C., Zhang X., Bie J., Kang W., Sun G., Zhao Q., Li L., Hu Q. (2026). Gut microbiota drives dietary lignans to improve perimenopausal depression via activating hippocampal ERβ/GluN2A/PSD95 pathway. Pharmacol. Res..

[61] Zheng K.Y., Gao B., Wang H.J., He J.G., Chen H.S., Hu Z.L., Long L.H., Chen J.G., Wang F. (2024). Melatonin Ameliorates Depressive-Like Behaviors in Ovariectomized Mice by Improving Tryptophan Metabolism via Inhibition of Gut Microbe Alistipes Inops. Adv. Sci..

[62] de Souza S.L., Aparecida Gomes D., da Silva C.M., Barros W.M.A., Alves S.M., Manhães de Castro R. (2026). Tryptophan Metabolism in Developmental Origins of Health and Disease. Nutr. Rev..

[63] Villani S., Fallarini S., Rezzi S.J., Di Martino R.M.C., Aprile S., Del Grosso E. (2024). Selective inhibition of indoleamine and tryptophan 2,3-dioxygenases: Comparative study on kynurenine pathway in cell lines via LC-MS/MS-based targeted metabolomics. J. Pharm. Biomed. Anal..

[64] Badawy A.A., Dawood S. (2025). Molecular Insights Into the Interaction of Tryptophan Metabolites with Tryptophan and Indoleamine 2,3-Dioxygenases: Nitric Oxide a New Effector of Tryptophan 2,3-Dioxygenase and Their Roles in Infection. Int. J. Tryptophan Res. IJTR.

[65] Tiwari S., Paramanik V. (2025). Role of Probiotics in Depression: Connecting Dots of Gut-Brain-Axis Through Hypothalamic-Pituitary Adrenal Axis and Tryptophan/Kynurenic Pathway involving Indoleamine-2,3-dioxygenase. Mol. Neurobiol..

[66] Alcantara-Zapata D.E., Lucero N., De Gregorio N., Astudillo Cornejo P., Ibarra Villanueva C., Baltodano-Calle M.J., Gonzales G.F., Behn C. (2022). Women’s mood at high altitude. sexual dimorphism in hypoxic stress modulation by the tryptophan-melatonin axis. Front. Physiol..

[67] Badawy A.A., Dawood S., Bano S. (2023). Kynurenine pathway of tryptophan metabolism in pathophysiology and therapy of major depressive disorder. World J. Psychiatry.

[68] Zhang K., Lei N., Li M., Li J., Li C., Shen Y., Guo P., Xiong L., Xie Y. (2021). Cang-Ai Volatile Oil Ameliorates Depressive Behavior Induced by Chronic Stress Through IDO-Mediated Tryptophan Degradation Pathway. Front. Psychiatry.

[69] Turek J., Gąsior Ł. (2023). Estrogen fluctuations during the menopausal transition are a risk factor for depressive disorders. Pharmacol. Rep. PR.

[70] McLaren S., Seidler K., Neil J. (2024). Investigating the Role of 17β-Estradiol on the Serotonergic System, Targeting Soy Isoflavones as a Strategy to Reduce Menopausal Depression: A Mechanistic Review. J. Am. Nutr. Assoc..

[71] Nagao R., Kawabata K., Mizutani Y., Shima S., Ueda A., Ito M., Maeda Y., Mouri A., Watanabe H. (2026). Altered Cerebrospinal Fluid Tryptophan-Kynurenine Pathway Metabolism in Multiple System Atrophy. Mov. Disord. Off. J. Mov. Disord. Soc..

[72] Kaur M., Porel P., Patel R., Aran K.R. (2025). Kynurenine Pathway in Epilepsy: Unraveling Its Role in Glutamate Excitotoxicity, GABAergic Dysregulation, Neuroinflammation, and Mitochondrial Dysfunction. Neurotox. Res..

[73] Liu J., Si J., Zhao W. (2025). Investigation of the Effect of Tai Chi Training on Depressive Symptoms in Perimenopausal Women on the Basis of Serum Kynurenine Metabolites. Exp. Aging Res..

[74] Sinder S.B., Sharma S.V., Shirvaikar I.S., Pradhyumnan H., Patel S.H., Cabeda Diaz I., Perez G.G., Bramlett H.M., Raval A.P. (2024). Impact of menopause-associated frailty on traumatic brain injury. Neurochem. Int..

[75] Ye M.S., Lee S.C., Kim S.K., Shim I. (2025). Evaluation of antidepressant and sleep-promoting effects of cordycepin in a menopause-like stress model. Biomed. Pharmacother. Biomed. Pharmacother..

[76] Ma J., Guo C.Y., Li H.B., Wu S.H., Li G.L. (2023). Prophylactic Effects of Hemp Seed Oil on Perimenopausal Depression: A Role of HPA Axis. J. Oleo Sci..

[77] Solimena M., De Camilli P. (1991). Autoimmunity to glutamic acid decarboxylase (GAD) in Stiff-Man syndrome and insulin-dependent diabetes mellitus. Trends Neurosci..

[78] Turský T. (1993). Gamma-aminobutyric acid and glutamate decarboxylase. Bratisl. Lek. Listy.

[79] Strandwitz P. (2018). Neurotransmitter modulation by the gut microbiota. Brain Res..

[80] Chen M., Ruan G., Chen L., Ying S., Li G., Xu F., Xiao Z., Tian Y., Lv L., Ping Y. (2022). Neurotransmitter and Intestinal Interactions: Focus on the Microbiota-Gut-Brain Axis in Irritable Bowel Syndrome. Front. Endocrinol..

[81] Deng Y., Zhou M., Wang J., Yao J., Yu J., Liu W., Wu L., Wang J., Gao R. (2021). Involvement of the microbiota-gut-brain axis in chronic restraint stress: Disturbances of the kynurenine metabolic pathway in both the gut and brain. Gut Microbes.

[82] Antonelli A., Giannini A., Chedraui P., Monteleone P., Caretto M., Genazzani A.D., Mannella P., Simoncini T., Genazzani A.R. (2022). Mood disorders and hormonal status across women’s life: A narrative review. Gynecol. Endocrinol. Off. J. Int. Soc. Gynecol. Endocrinol..

[83] Czéh B., Vardya I., Varga Z., Febbraro F., Csabai D., Martis L.S., Højgaard K., Henningsen K., Bouzinova E.V., Miseta A. (2018). Long-Term Stress Disrupts the Structural and Functional Integrity of GABAergic Neuronal Networks in the Medial Prefrontal Cortex of Rats. Front. Cell. Neurosci..

[84] Tran K.H., Luki J., Hanstock S., Hanstock C.C., Seres P., Aitchison K., Shandro T., Le Melledo J.M. (2023). Decreased GABA+ Levels in the Medial Prefrontal Cortex of Perimenopausal Women: A 3T 1H-MRS Study. Int. J. Neuropsychopharmacol..

[85] Fogaça M.V., Duman R.S. (2019). Cortical GABAergic Dysfunction in Stress and Depression: New Insights for Therapeutic Interventions. Front. Cell. Neurosci..

[86] Proserpio P., Marra S., Campana C., Agostoni E.C., Palagini L., Nobili L., Nappi R.E. (2020). Insomnia and menopause: A narrative review on mechanisms and treatments. Climacteric J. Int. Menopause Soc..

[87] Antonoudiou P., Tan Y.L., Kontou G., Upton A.L., Mann E.O. (2020). Parvalbumin and Somatostatin Interneurons Contribute to the Generation of Hippocampal Gamma Oscillations. J. Neurosci. Off. J. Soc. Neurosci..

[88] Cho J.M., Lee J., Ahn E.M., Bae J. (2025). Beyond Hot Flashes: The Role of Estrogen Receptors in Menopausal Mental Health and Cognitive Decline. Brain Sci..

[89] Gordon J.L., Eisenlohr-Moul T.A., Rubinow D.R., Schrubbe L., Girdler S.S. (2016). Naturally Occurring Changes in Estradiol Concentrations in the Menopause Transition Predict Morning Cortisol and Negative Mood in Perimenopausal Depression. Clin. Psychol. Sci. A J. Assoc. Psychol. Sci..

[90] Liu Z.P., Song C., Wang M., He Y., Xu X.B., Pan H.Q., Chen W.B., Peng W.J., Pan B.X. (2014). Chronic stress impairs GABAergic control of amygdala through suppressing the tonic GABAA receptor currents. Mol. Brain.

[91] Luscher B., Shen Q., Sahir N. (2011). The GABAergic deficit hypothesis of major depressive disorder. Mol. Psychiatry.

[92] Barrett E., Ross R.P., O’Toole P.W., Fitzgerald G.F., Stanton C. (2012). Γ-Aminobutyric acid production by culturable bacteria from the human intestine. J. Appl. Microbiol..

[93] Bravo J.A., Forsythe P., Chew M.V., Escaravage E., Savignac H.M., Dinan T.G., Bienenstock J., Cryan J.F. (2011). Ingestion of Lactobacillus strain regulates emotional behavior and central GABA receptor expression in a mouse via the vagus nerve. Proc. Natl. Acad. Sci. USA.

[94] Kelly J.R., Kennedy P.J., Cryan J.F., Dinan T.G., Clarke G., Hyland N.P. (2015). Breaking down the barriers: The gut microbiome, intestinal permeability and stress-related psychiatric disorders. Front. Cell. Neurosci..

[95] Gordon J.L., Girdler S.S., Meltzer-Brody S.E., Stika C.S., Thurston R.C., Clark C.T., Prairie B.A., Moses-Kolko E., Joffe H., Wisner K.L. (2015). Ovarian hormone fluctuation, neurosteroids, and HPA axis dysregulation in perimenopausal depression: A novel heuristic model. Am. J. Psychiatry.

[96] Birkhäuser M. (2021). Climacteric depression and anxiety. Ther. Umsch. Rev. Ther..

[97] Merino Del Portillo M., Clemente-Suárez V.J., Ruisoto P., Jimenez M., Ramos-Campo D.J., Beltran-Velasco A.I., Martínez-Guardado I., Rubio-Zarapuz A., Navarro-Jiménez E., Tornero-Aguilera J.F. (2024). Nutritional Modulation of the Gut-Brain Axis: A Comprehensive Review of Dietary Interventions in Depression and Anxiety Management. Metabolites.

[98] Zamoscik V., Schmidt S.N.L., Bravo R., Ugartemendia L., Plieger T., Rodríguez A.B., Reuter M., Kirsch P. (2021). Tryptophan-enriched diet or 5-hydroxytryptophan supplementation given in a randomized controlled trial impacts social cognition on a neural and behavioral level. Sci. Rep..

[99] Yousefzadeh S.A., Jarah M., Riazi G.H. (2020). Tryptophan Improves Memory Independent of Its Role as a Serotonin Precursor: Potential Involvement of Microtubule Proteins. J. Mol. Neurosci. MN.

[100] Reuter M., Zamoscik V., Plieger T., Bravo R., Ugartemendia L., Rodriguez A.B., Kirsch P. (2021). Tryptophan-rich diet is negatively associated with depression and positively linked to social cognition. Nutr. Res..

[101] Meloni M., Puligheddu M., Carta M., Cannas A., Figorilli M., Defazio G. (2020). Efficacy and safety of 5-hydroxytryptophan on depression and apathy in Parkinson’s disease: A preliminary finding. Eur. J. Neurol..

[102] Li S., Sutanto C.N., Xia X., Kim J.E. (2025). The Impact of 5-Hydroxytryptophan Supplementation on Cognitive Function and Mood in Singapore Older Adults: A Randomized Controlled Trial. Nutrients.

[103] Nash E., Jamshidi N. (2022). Hippocampal ischaemia from accidental 5-Hydroxytryptophan (5-HTP) overdose case report. Clin. Neurol. Neurosurg..

[104] Izumi T., Iwamoto N., Kitaichi Y., Kato A., Inoue T., Koyama T. (2007). Effects of co-administration of antidepressants and monoamine oxidase inhibitors on 5-HT-related behavior in rats. Eur. J. Pharmacol..

[105] Yamamoto R., Yamamoto Y., Imai S., Fukutomi R., Ozawa Y., Abe M., Matuo Y., Saito K. (2014). Effects of various phytochemicals on indoleamine 2,3-dioxygenase 1 activity: Galanal is a novel, competitive inhibitor of the enzyme. PLoS ONE.

[106] Jeong Y.I., Kim S.W., Jung I.D., Lee J.S., Chang J.H., Lee C.M., Chun S.H., Yoon M.S., Kim G.T., Ryu S.W. (2009). Curcumin suppresses the induction of indoleamine 2,3-dioxygenase by blocking the Janus-activated kinase-protein kinase Cdelta-STAT1 signaling pathway in interferon-gamma-stimulated murine dendritic cells. J. Biol. Chem..

[107] Noh K.T., Chae S.H., Chun S.H., Jung I.D., Kang H.K., Park Y.M. (2013). Resveratrol suppresses tumor progression via the regulation of indoleamine 2,3-dioxygenase. Biochem. Biophys. Res. Commun..

[108] Ebokaiwe A.P., Ushang O.R., Ogunwa T.H., Kikiowo B., Olusanya O. (2022). Quercetin attenuates cyclophosphamide induced-immunosuppressive indoleamine 2,3-dioxygenase in the hippocampus and cerebral cortex of male Wister rats. J. Biochem. Mol. Toxicol..

[109] Liu X.C., Erhardt S., Goiny M., Engberg G., Mathé A.A. (2017). Decreased levels of kynurenic acid in prefrontal cortex in a genetic animal model of depression. Acta Neuropsychiatr..

[110] Liu B.B., Luo L., Liu X.L., Geng D., Liu Q., Yi L.T. (2015). 7-Chlorokynurenic acid (7-CTKA) produces rapid antidepressant-like effects: Through regulating hippocampal microRNA expressions involved in TrkB-ERK/Akt signaling pathways in mice exposed to chronic unpredictable mild stress. Psychopharmacology.

[111] Li C.F., Chen X.M., Chen S.M., Mu R.H., Liu B.B., Luo L., Liu X.L., Geng D., Liu Q., Yi L.T. (2016). Activation of hippocampal BDNF signaling is involved in the antidepressant-like effect of the NMDA receptor antagonist 7-chlorokynurenic acid. Brain Res..

[112] Majewski M., Kozlowska A., Thoene M., Lepiarczyk E., Grzegorzewski W.J. (2016). Overview of the role of vitamins and minerals on the kynurenine pathway in health and disease. J. Physiol. Pharmacol. Off. J. Pol. Physiol. Soc..

[113] Rios-Avila L., Nijhout H.F., Reed M.C., Sitren H.S., Gregory J.F. (2013). A mathematical model of tryptophan metabolism via the kynurenine pathway provides insights into the effects of vitamin B-6 deficiency, tryptophan loading, and induction of tryptophan 2,3-dioxygenase on tryptophan metabolites. J. Nutr..

[114] Paiardini A., Giardina G., Rossignoli G., Voltattorni C.B., Bertoldi M. (2017). New Insights Emerging from Recent Investigations on Human Group II Pyridoxal 5′-Phosphate Decarboxylases. Curr. Med. Chem..

[115] Midttun O., Ulvik A., Ringdal Pedersen E., Ebbing M., Bleie O., Schartum-Hansen H., Nilsen R.M., Nygård O., Ueland P.M. (2011). Low plasma vitamin B-6 status affects metabolism through the kynurenine pathway in cardiovascular patients with systemic inflammation. J. Nutr..

[116] Liaquat M., Minihane A.M., Vauzour D., Pontifex M.G. (2025). The gut microbiota in menopause: Is there a role for prebiotic and probiotic solutions?. Post. Reprod. Health.

[117] Marano G., Traversi G., Mazza O., Caroppo E., Capristo E., Gaetani E., Mazza M. (2025). The Immune Mind: Linking Dietary Patterns, Microbiota, and Psychological Health. Nutrients.

[118] Liu G., Chong H.X., Chung F.Y., Li Y., Liong M.T. (2020). Lactobacillus plantarum DR7 Modulated Bowel Movement and Gut Microbiota Associated with Dopamine and Serotonin Pathways in Stressed Adults. Int. J. Mol. Sci..

[119] Chong H.X., Yusoff N.A.A., Hor Y.Y., Lew L.C., Jaafar M.H., Choi S.B., Yusoff M.S.B., Wahid N., Abdullah M., Zakaria N. (2019). Lactobacillus plantarum DR7 alleviates stress and anxiety in adults: A randomised, double-blind, placebo-controlled study. Benef. Microbes.

[120] Zhang K., Chen L., Yang J., Liu J., Li J., Liu Y., Li X., Chen L., Hsu C., Zeng J. (2023). Gut microbiota-derived short-chain fatty acids ameliorate methamphetamine-induced depression- and anxiety-like behaviors in a Sigmar-1 receptor-dependent manner. Acta Pharm. Sin. B.

[121] Davani-Davari D., Negahdaripour M., Karimzadeh I., Seifan M., Mohkam M., Masoumi S.J., Berenjian A., Ghasemi Y. (2019). Prebiotics: Definition, Types, Sources, Mechanisms, and Clinical Applications. Foods.

[122] Markowiak-Kopeć P., Śliżewska K. (2020). The Effect of Probiotics on the Production of Short-Chain Fatty Acids by Human Intestinal Microbiome. Nutrients.

[123] Bourassa M.W., Alim I., Bultman S.J., Ratan R.R. (2016). Butyrate, neuroepigenetics and the gut microbiome: Can a high fiber diet improve brain health?. Neurosci. Lett..

[124] Cheng J., Hu H., Ju Y., Liu J., Wang M., Liu B., Zhang Y. (2024). Gut microbiota-derived short-chain fatty acids and depression: Deep insight into biological mechanisms and potential applications. General. Psychiatry.

[125] Ventriglio A., Sancassiani F., Contu M.P., Latorre M., Di Salvatore M., Fornaro M., Bhugra D. (2020). Mediterranean Diet and its Benefits on Health and Mental Health: A Literature Review. Clin. Pract. Epidemiol. Ment. Health CP EMH.

[126] Su Y., Xia Y. (2026). Gut microbiota dysbiosis and depression: Bidirectional interactions, mediating pathways, and microecological therapeutics. Curr. Res. Food Sci..

[127] Hepsomali P., Groeger J.A., Nishihira J., Scholey A. (2020). Effects of Oral Gamma-Aminobutyric Acid (GABA) Administration on Stress and Sleep in Humans: A Systematic Review. Front. Neurosci..

[128] Bartholini G. (1985). GABA receptor agonists: Pharmacological spectrum and therapeutic actions. Med. Res. Rev..

[129] Baek J.H., Park H., Kang H., Kim R., Kang J.S., Kim H.J. (2024). The Role of Glutamine Homeostasis in Emotional and Cognitive Functions. Int. J. Mol. Sci..

[130] Young L.S., Bye R., Scheltinga M., Ziegler T.R., Jacobs D.O., Wilmore D.W. (1993). Patients receiving glutamine-supplemented intravenous feedings report an improvement in mood. JPEN. J. Parenter. Enter. Nutr..

[131] Jia F., Yue M., Chandra D., Keramidas A., Goldstein P.A., Homanics G.E., Harrison N.L. (2008). Taurine is a potent activator of extrasynaptic GABA(A) receptors in the thalamus. J. Neurosci. Off. J. Soc. Neurosci..

[132] Zhu Y., Wang R., Fan Z., Luo D., Cai G., Li X., Han J., Zhuo L., Zhang L., Zhang H. (2023). Taurine Alleviates Chronic Social Defeat Stress-Induced Depression by Protecting Cortical Neurons from Dendritic Spine Loss. Cell. Mol. Neurobiol..

[133] Fenalti G., Law R.H., Buckle A.M., Langendorf C., Tuck K., Rosado C.J., Faux N.G., Mahmood K., Hampe C.S., Banga J.P. (2007). GABA production by glutamic acid decarboxylase is regulated by a dynamic catalytic loop. Nat. Struct. Mol. Biol..

[134] Williams M.J., Harris R.I., Dean B.C. (1985). Controlled trial of pyridoxine in the premenstrual syndrome. J. Int. Med. Res..

[135] Durrani D., Idrees R., Idrees H., Ellahi A. (2022). Vitamin B6: A new approach to lowering anxiety, and depression?. Ann. Med. Surg..

[136] Sartori S.B., Whittle N., Hetzenauer A., Singewald N. (2012). Magnesium deficiency induces anxiety and HPA axis dysregulation: Modulation by therapeutic drug treatment. Neuropharmacology.

[137] Moabedi M., Aliakbari M., Erfanian S., Milajerdi A. (2023). Magnesium supplementation beneficially affects depression in adults with depressive disorder: A systematic review and meta-analysis of randomized clinical trials. Front. Psychiatry.

[138] Boyle N.B., Lawton C., Dye L. (2017). The Effects of Magnesium Supplementation on Subjective Anxiety and Stress-A Systematic Review. Nutrients.

[139] Hidese S., Ogawa S., Ota M., Ishida I., Yasukawa Z., Ozeki M., Kunugi H. (2019). Effects of L-Theanine Administration on Stress-Related Symptoms and Cognitive Functions in Healthy Adults: A Randomized Controlled Trial. Nutrients.

[140] Bulman A., D’Cunha N.M., Marx W., Turner M., McKune A., Naumovski N. (2025). The effects of L-theanine consumption on sleep outcomes: A systematic review and meta-analysis. Sleep Med. Rev..

[141] Matta T., Bhatia R., Joshi S.R., Bishnoi M., Chopra K., Kondepudi K.K. (2024). GABA synthesizing lactic acid bacteria and genomic analysis of Levilactobacillus brevis LAB6. Biotech.

[142] Cataldo P.G., Urquiza Martínez M.P., Villena J., Kitazawa H., Saavedra L., Hebert E.M. (2024). Comprehensive characterization of γ-aminobutyric acid (GABA) production by Levilactobacillus brevis CRL 2013: Insights from physiology, genomics, and proteomics. Front. Microbiol..

[143] Pokusaeva K., Johnson C., Luk B., Uribe G., Fu Y., Oezguen N., Matsunami R.K., Lugo M., Major A., Mori-Akiyama Y. (2017). GABA-producing Bifidobacterium dentium modulates visceral sensitivity in the intestine. Neurogastroenterol. Motil..

[144] Luck B., Horvath T.D., Engevik K.A., Ruan W., Haidacher S.J., Hoch K.M., Oezguen N., Spinler J.K., Haag A.M., Versalovic J. (2021). Neurotransmitter Profiles Are Altered in the Gut and Brain of Mice Mono-Associated with Bifidobacterium dentium. Biomolecules.

[145] Dahiya D., Nigam P.S. (2022). Nutrition and Health through the Use of Probiotic Strains in Fermentation to Produce Non-Dairy Functional Beverage Products Supporting Gut Microbiota. Foods.

[146] Del Toro-Barbosa M., Uribe-Velázquez T., Hurtado-Romero A., Rosales-De la Cruz M.F., Carrillo-Nieves D., Garcia-Amezquita L.E., García-Cayuela T. (2025). Evaluation of GABA-Producing Fermented Whey Formulations: From Strain Selection to Raspberry-Enriched Beverages with Psychobiotic Potential. Foods.

[147] Scarpelin C., de Souza Cordes C.L., Kamimura E.S., Macedo J.A., de Paula Menezes Barbosa P., Alves Macedo G. (2025). New plant-based kefir fermented beverages as potential source of GABA. J. Food Sci. Technol..

[148] Mei Z., Yuan J., Li D. (2022). Biological activity of galacto-oligosaccharides: A review. Front. Microbiol..

[149] Richards P.J., Almutrafy A., Liang L., Flaujac Lafontaine G.M., King E., Fish N.M., Connerton A.J., Connerton P.L., Connerton I.F. (2024). Prebiotic galactooligosaccharide feed modifies the chicken gut microbiota to efficiently clear Salmonella. mSystems.

[150] Ng Q.X., Lim Y.L., Yaow C.Y.L., Ng W.K., Thumboo J., Liew T.M. (2023). Effect of Probiotic Supplementation on Gut Microbiota in Patients with Major Depressive Disorders: A Systematic Review. Nutrients.

